# Ultrasound *omental* fat as a precocious marker of metabolic adiposity in children

**DOI:** 10.3389/fped.2026.1830258

**Published:** 2026-05-28

**Authors:** Daniel Sánchez-Ruiz, Mònica Peitx, Marta Calbo, Olga Rossell, Laia Dachs, Júlia Bonet, Andrea Jover, Guillem Cuatrecasas

**Affiliations:** 1 Grup CPEN Endocrinologia I Nutrició, Barcelona, Spain; 2 Unidad Multidisciplinaria de Obesidad Hospital Teknon, Grupo Quiron Salud, Barcelona, Spain; 3 Faculty Health Sciences, Open University Catalonia, Barcelona, Spain

**Keywords:** abdominal ultrasound, *Eco-Obesity*, *omental* fat, pediatric obesity, visceral adiposity

## Abstract

**Background:**

Visceral adiposity in pediatric obesity is conventionally assessed through aggregate measures that lack compartmental resolution. The *Eco-Obesity* ultrasound protocol enables simultaneous quantification of distinct abdominal fat layers. This study aims to evaluate the clinical and metabolic correlates of each compartment in a pediatric cohort with overweight or obesity.

**Methods:**

Retrospective cross-sectional study of consecutive pediatric patients evaluated at a multidisciplinary obesity unit. All underwent structured *Eco-Obesity* ultrasound, anthropometric assessment, bioelectrical impedance, and fasting biochemical profiling. Pearson correlations between fat layer thickness and cardiometabolic parameters were computed for the overall cohort and stratified by sex. ROC curve analysis was performed to identify optimal *omental* fat thickness cut-offs for the discrimination of metabolically unhealthy status, defined as the fulfilment of at least one criterion: HDL-cholesterol < 40 mg/dL, triglycerides > 150 mg/dL, fasting glucose > 100 mg/dL, blood pressure above the 90th percentile, HOMA-IR > 3.16, or hepatic steatosis.

**Results:**

The cohort comprised 84 pediatric patients (62% female; mean age 14.0 ± 2.6 years; mean BMI SDS 2.8 ± 1.1), of whom 71.3% were classified as obese (class I–III) and 28.7% as overweight with abdominal obesity; 28.3% presented ultrasound-diagnosed hepatic steatosis. *Omental* fat was the only compartment consistently associated with anthropometric obesity indices, insulin resistance, LDL cholesterol, and hepatic steatosis, and the only one to exhibit significant sexual dimorphism (40.5 mm males vs. 27.3 mm females; *p* = <0.001). Associations were markedly stronger in males. ROC analysis to predict metabolic syndrome showed AUCs of 0.64 (males; cut-off 34.6 mm) and 0.57 (females; cut-off 19.6 mm).

**Conclusion:**

*Omental* fat appears to be the visceral compartment most consistently associated with metabolic risk markers in pediatric obesity, with sex-specific thresholds substantially lower than adult reference values, reinforcing the need for a dedicated pediatric cut-off.

## 1 Introduction

Childhood and adolescent obesity are among the most pressing public health challenges of the twenty-first century (1), with prevalence rates that have increased over the past four decades across all geographic regions and socioeconomic groups and is the basis for the huge rise of adult obesity rates (2). Beyond the well-characterized increase in total fat mass, recent evidence indicates that the body distribution of adipose tissue plays a decisive role in determining cardiometabolic risk from early life stages. In adults, the pathogenic contribution of specific visceral fat compartments, including *omental*, *perirenal*, and *epicardial* adipose tissue, to the development of insulin resistance, type 2 diabetes, dyslipidemia, hepatic steatosis, and atherosclerotic cardiovascular disease has been extensively documented (3, 4). However, the characterization of these distinct visceral fat depots and their metabolic correlates in pediatric populations remains remarkably underexplored, limiting the capacity of clinicians to implement precise and early risk stratification strategies in children and adolescents with excess adiposity.

Conventional approaches to body composition assessment in pediatric obesity have traditionally relied on anthropometric surrogates such as body mass index (BMI), waist circumference, and the waist-to-height ratio, or on instrumental techniques including bioelectrical impedance analysis and, less frequently, dual-energy x-ray absorptiometry (DEXA). These methods provide valuable estimates of total and regional adiposity, but they share a fundamental limitation: the inability to discriminate between the individual visceral fat layers that have been associated with differential metabolic activity and divergent associations with cardiometabolic risk. DEXA-derived visceral adipose tissue (VAT) quantification, for instance, represents an aggregate measure corresponding to the sum of *preperitoneal*, *omental*, and *perirenal* compartments, without the resolution to assess each depot independently (5). This distinction is clinically relevant, as evidence from adult cohorts has consistently demonstrated that not all visceral fat compartments carry equivalent metabolic significance (5, 6).

The *Eco-Obesity* ultrasound protocol, originally developed and validated in adult populations with obesity, is a non-invasive, radiation-free technique that enables the simultaneous quantification of multiple abdominal fat layers with metabolic and cardiovascular relevance: *preperitoneal*, *omental*, *perirenal* and *epicardial* fat layers (7). In a recent cross-sectional study of 402 adults with obesity, di Gregorio et al. (5) demonstrated that *omental* and *perirenal* fat layers, but not *preperitoneal* fat, correlated significantly with anthropometric indices, DEXA-derived VAT, and metabolic parameters including fasting glycemia, HOMA-IR, and the lipid profile, challenging the assumption that *preperitoneal* fat could serve as a surrogate for visceral adiposity. The same study integrated echocardiographic assessment of epicardial adipose tissue (EAT) within the *Eco-Obesity* protocol, presenting significant correlations between this metabolically active cardiac depot and both *omental* and *perirenal* fat layers, DEXA-derived VAT, and biochemical markers of insulin resistance and dyslipidemia.

Despite this robust body of evidence in adults, the applicability of structured ultrasound evaluation of abdominal fat layers to pediatric populations remains largely unexplored. The identification of which specific visceral fat depots become pathologically expanded in pediatric obesity, and at what stage of adiposity progression, could inform both the understanding of the natural history of ectopic fat deposition and the selection of appropriate monitoring and therapeutic strategies. Of particular interest is whether the pattern of visceral fat accumulation observed in adults is already discernible in the pediatric age range, or whether a sequential pattern of fat depot expansion exists in which certain compartments are affected earlier than others.

In the present study we aimed to evaluate the different abdominal fat layers by structured *Eco-Obesity* ultrasound in a cohort of pediatric patients with obesity or overweight with abdominal obesity, and to establish their associations with anthropometric parameters, body fat percentage, markers of insulin resistance, the lipid profile, and the presence of hepatic steatosis. Secondary objectives included the comparison of fat layer thickness between overweight and obese subgroups, the assessment of sexual dimorphism in fat depot distribution, and the evaluation of whether sex-stratified correlation analysis could unmask stronger associations between *omental* fat and cardiometabolic risk parameters, and the determination of sex-specific optimal *omental* fat thickness cut-off values for the discrimination of metabolically unhealthy status through receiver operating characteristic (ROC) curve analysis.

## 2 Methods

### 2.1 Study design and setting

Retrospective, cross-sectional, descriptive study of consecutive patients evaluated at the Pediatric Endocrinology outpatient clinic between 2023 and 2025. Patients were referred for assessment of excess adiposity and associated metabolic comorbidities as part of routine clinical care.

Inclusion criteria comprised: age between 6 and 18 years and a diagnosis of obesity or overweight, accompanied by abdominal obesity, defined as a waist-to-height ratio ≥ 0.5 (8). Overweight and obesity were defined as a BMI ≥ 1 standard deviation above the age- and sex-specific median (≥ 85th percentile) and a BMI ≥ 2 standard deviations above the age- and sex-specific median (≥ 95th percentile), respectively, according to the Endocrine Society Clinical Practice Guideline (9). Obesity was further subclassified into class I [BMI between 2.0 and 2.5 standard deviation (SD), < 120% of the 95th percentile], class II or severe (BMI between 2.5 and 3.5 SD, 120%–140% of the 95th percentile), and class III or morbid (BMI > 3.5 SD, > 140% of the 95th percentile) (9, 10). Exclusion criteria included secondary causes of obesity (endocrine disorders such as hypothyroidism, Cushing syndrome, growth hormone deficiency, genetic syndromes associated with obesity), use of medications known to affect body composition or lipid metabolism (corticosteroids, antipsychotics), and the presence of chronic systemic diseases that could independently alter fat distribution or metabolic parameters.

Pubertal development was assessed at the time of clinical evaluation by a trained pediatric endocrinologist using the Tanner staging system (11). The overall Tanner stage assigned to each patient corresponded to the composite clinical assessment performed during the physical examination as part of the routine endocrinological evaluation. Tanner stage I was considered indicative of prepubertal status, stages II through IV as indicative of active pubertal progression, and stage V as denoting completion of pubertal development.

All patients underwent a complete clinical evaluation that included detailed anthropometric measurements, analysis of biochemical parameters and a structured abdominal ultrasound assessment of fat layers following the *Eco-Obesity* protocol.

### 2.2 Anthropometric measurements

Standardized anthropometric measurements were performed by trained personnel following international protocols. Body weight (kg) was measured with a calibrated digital scale with subjects wearing light clothing and no shoes. Height (cm) was measured using a wall-mounted stadiometer. BMI was calculated as weight (kg) divided by height squared (m^2^).

Total body fat was measured by multipolar bioelectrical impedanciometry (Inbody 530©), using 15 different measures (30-s assessment) at three different frequencies (5-50-500 kHz) in left arm, right arm, trunk, left leg and right leg. Measurements were performed under standardized conditions: subjects fasted for at least 2 h, voided before the test, stood barefoot on the platform electrodes, and grasped the hand electrodes with arms slightly abducted from the trunk.

### 2.3 Biochemical parameters

Biochemical parameters were obtained from fasting venous blood samples drawn after a minimum 10-hour overnight fast. The analytical panel included fasting plasma glucose (mg/dL), fasting serum insulin (µU/mL), and the calculation of the homeostatic model assessment for insulin resistance (HOMA-IR), computed as [fasting glucose (mg/dL) × fasting insulin (µU/mL)]/405. Lipid profiling included total cholesterol, high-density lipoprotein cholesterol (HDL, mg/dL), low-density lipoprotein cholesterol (LDL, mg/dL), and triglycerides (TG, mg/dL). All biochemical determinations were performed following standardized laboratory protocols. Insulin was measured by enzyme immunoassay using ALINITY C reagents© (Abbott Diagnostics) with a sensitivity of 3 mU/mL.

### 2.4 Structured abdominal ultrasound assessment of fat layers

All participants underwent a standardized abdominal ultrasound evaluation following the *Eco-Obesity* protocol originally described by Cuatrecasas et al. (7) in adult populations and adapted for the pediatric age range. Examinations were performed using a MyLab Gamma ultrasound system (Esaote S.p.A.). All ultrasound examinations were performed by a single experienced operator (with over 15 years of experience in abdominal ultrasound and body composition assessment) following a standardized protocol to minimize intra-observer variability. The intra-observer reproducibility of the *Eco-Obesity* protocol has been previously documented in adult populations (7, 12). A formal assessment of intra-observer variability in the present pediatric cohort was not performed, and the operator was not formally blinded to the patients’ clinical or biochemical data; these limitations should be considered when interpreting the results. Examinations were performed with the patient in the supine position and during suspended respiration at end-expiration. All five fat layer measurements were obtained at a single standardized anatomical landmark, defined as the level of the bifurcation of the abdominal aorta into the common iliac arteries, which corresponds ecographically to the L4 vertebral level; this reference point aligns with the anatomical plane conventionally used for visceral fat quantification by DEXA and computed tomography, thereby facilitating methodological comparability with reference body composition imaging techniques. Compared to CT and MRI, which remain the gold standard for visceral fat quantification but are limited in pediatric populations by radiation exposure, cost, and accessibility, the *Eco-Obesity* ultrasound protocol offers a radiation-free, portable, and repeatable alternative suitable for clinical screening and longitudinal monitoring in this age group. (i) superficial *subcutaneous* fat, defined as the adipose layer between the skin and the superficial fascia (fascia of Camper); (ii) deep *subcutaneous* fat, measured between the superficial fascia and the external oblique muscle aponeurosis; (iii) *preperitoneal* fat, corresponding to the adipose layer located between the internal surface of the rectus abdominis muscle and the anterior peritoneal surface; (iv) *omental* fat, measured as the distance from the anterior peritoneal wall to the anterior surface of the aorta, encompassing the *omental* and mesenteric adipose compartment; and (v) right *perirenal* fat, measured as the maximal thickness of the adipose capsule between the renal cortex and the inner margin of the abdominal wall musculature in the right posterior pararenal space. All thickness measurements were obtained perpendicularly to the tissue planes, with the transducer held with minimal pressure to avoid compression of the subcutaneous layers. Each measurement was recorded as the mean of three consecutive acquisitions.

The presence and degree of hepatic steatosis were evaluated as part of the structured abdominal ultrasound examination performed following the *Eco-Obesity* protocol. The diagnosis and grading were based on standard criteria, including increased hepatic echogenicity relative to the renal cortex, posterior attenuation of the ultrasound beam, and loss of definition of the intrahepatic vascular structures and the diaphragm. Steatosis was graded as absent, mild (grade I: slight increase in hepatic echogenicity with normal visualization of the diaphragm and portal vein walls), moderate (grade II: moderate increase in echogenicity with slightly impaired visualization of portal vein walls and the diaphragm), or severe (grade III: marked increase in echogenicity with poor or absent visualization of the diaphragm, posterior portion of the right hepatic lobe, and portal vein walls) (13).

### 2.5 Statistical analyses

Statistical analyses were performed using SPSS version 19 (IBM Corp., Armonk, NY, USA). The normality of continuous variables was assessed using the Shapiro–Wilk test. Continuous variables are expressed as mean ± SD, and categorical variables as absolute frequencies and percentages. Comparisons of mean fat layer thickness between two groups (males vs. females; overweight vs. obesity; presence vs. absence of hepatic steatosis) were performed using the independent-samples Student's t-test for normally distributed variables. Pearson correlation coefficients (R) were calculated to assess linear associations between ultrasound-measured fat layer thickness and anthropometric (BMI, WHtR, body fat percentage) and biochemical (HOMA-IR, LDL, HDL, triglycerides) variables. To evaluate whether sexual dimorphism influenced the strength of these associations, correlation analyses were performed both for the overall cohort and stratified by sex. A *p* value < 0.05 was considered statistically significant for all analyses. To identify optimal *omental* fat thickness cut-off values for the discrimination of metabolically unhealthy status, defined as the fulfilment of at least one of the following criteria: fasting HDL-cholesterol < 40 mg/dL; fasting triglycerides > 150 mg/dL; fasting plasma glucose > 100 mg/dL; systolic or diastolic blood pressure above the 90th percentile for age, sex, and height; HOMA-IR > 3.16; or ultrasound-diagnosed hepatic steatosis, ROC curves were constructed for the overall cohort and for each sex separately. The area under the curve (AUC) was computed as the primary measure of discriminatory accuracy. Optimal cut-off values were determined by maximizing the Youden index (sensitivity + specificity - 1). The 95% confidence intervals (CI) of the optimal cut-off values were estimated by non-parametric bootstrap resampling (2,000 iterations).

### 2.6 Ethics statement

The study was conducted in accordance with the principles expressed in the Declaration of Helsinki, and the protocol followed the Good Clinical Practice guidelines and was approved by the Ethical Committee of Hospital Teknon-Grupo Quiron Salud, Barcelona. Given the retrospective nature of this study, which used data obtained from routine clinical practice, general informed consent for research use of anonymized clinical data was obtained from the parents or legal guardians of all participants. Assent was obtained from minors capable of understanding the nature of the study, in accordance with applicable local regulations.

## 3 Results

### 3.1 Demographic and clinical characteristics

A total of 84 pediatric patients were included in the analysis, of whom 52 (62%) were female and 32 (38%) were male. The mean age of the overall cohort was 14.0 ± 2.6 years, with females presenting a mean age of 14.3 ± 2.9 years and males 13.6 ± 2.2 years. With respect to pubertal staging according to Tanner criteria, most participants were in advanced pubertal development: 61.9% of the overall cohort had reached Tanner stage V, a proportion notably higher in females (78.8%) than in males (34.4%).

The mean BMI standard deviation score (SDS) for the overall cohort was 2.8 ± 1.1, with males presenting a numerically higher value than females. For obesity classification, 28.7% of participants were categorized as overweight with concurrent abdominal obesity, 19.0% as class I obesity (2.0–2.5 SD), 28.5% as class II obesity (2.5–3.5 SD), and 23.8% as class III obesity (>3.5 SD), with a similar distribution across sexes. The mean waist-to-height ratio was 0.58 ± 0.1 for the total sample, with similar values in males and females. Body fat percentage presented a mean of 43.4 ± 8.8% in the overall cohort, with slightly higher mean in females.

Regarding hepatic steatosis 71.7% of participants showed no evidence of hepatic fat deposition. Among those with steatosis, mild grade was the most prevalent (20.5%), followed by moderate steatosis (7.7%); no case of severe steatosis was identified in either sex group. Regarding the biochemical profile, males presented a mean HOMA-IR of 3.50 ± 2.42, mean triglycerides of 90.90 ± 39.51 mg/dL, mean LDL cholesterol of 96.48 ± 20.24 mg/dL, and mean HDL cholesterol of 48.67 ± 11.53 mg/dL. Females presented a mean HOMA-IR of 4.44 ± 5.36, mean triglycerides of 89.34 ± 41.52 mg/dL, mean LDL cholesterol of 102.65 ± 29.42 mg/dL, and mean HDL cholesterol of 47.71 ± 7.71 mg/dL. The descriptive characteristics of the cohort are summarized in Table 1.

**Table 1:** Descriptive characteristics of the population analyzed.

Variable	Overall (*n* = 84)	Female (*n* = 52)	Male (*n* = 32)
Sex			
Female	62%	100%	0%
Male	38%	0%	100%
Age (years, mean ± SD)	14.0 ± 2.6	14.3 ± 2.9	13.6 ± 2.2
Tanner stage			
Tanner I	14.3%	13.5%	15.6%
Tanner II	5.9%	1.9%	12.5%
Tanner III	10.7%	1.9%	25.0%
Tanner IV	7.2%	3.8%	12.5%
Tanner V	61.9%	78.8%	34.4%
BMI (SDS), mean ± SD	2.8 ± 1.1	2.6 ± 1.0	2.9 ± 1.2
Classification by BMI			
Overweight + abdominal obesity (waist-to-height > 0.5)	28.7%	28.9%	25.0%
Obesity I (2-2.5 SD)	19.0%	21.2%	15.6%
Obesity II (2.5–3.5 SD)	28.5%	26.9%	34.4%
Obesity III (>3.5 SD)	23.8%	23.1%	25.0%
Waist-to-height ratio, mean ± SD	0.58 ± 0.1	0.57 ± 0.1	0.60 ± 0.1
Body fat percentage (bioimpedance), mean ± SD	43.4 ± 8.8	44.7 ± 21.4	41.2 ± 19.6
Hepatic steatosis on ultrasound			
None	71.7%	76.9%	68.8%
Mild	20.5%	19.2%	18.8%
Moderate	7.7%	3.9%	12.5%
Severe	0.0%	0.0%	0.0%

### 3.2 Abdominal fat layer thickness by *Eco-Obesity* ultrasound

Thickness values for all five abdominal fat compartments measured by structured *Eco-Obesity* ultrasound, stratified by sex, weight status, and presence of hepatic steatosis, are presented in Table 2.

**Table 2:** Abdominal fat layer thickness (mm) measured by structured *Eco-Obesity* ultrasound according to sex, weight status, and presence of hepatic steatosis.

Group	Superficial subcutaneous fat	Deep subcutaneous fat	*Preperitoneal* fat	*Omental* fat	Right *perirenal* fat
Sex					
Female	12.7 (11.3–14.0)	16.0 (14.1–17.7)	7.4 (6.4–8.5)	27.3 (24.4–30.0)	8.0 (6.5–9.3)
Male	15.1 (12.8–17.4)	18.9 (16.0–21.6)	6.6 (5.3–7.8)	40.5 (35.3–45.6)	8.4 (6.4–10.2)
*P* value	0.070	0.172	0.303	<0.001	0.704
Weight status					
Overweight + abdominal obesity	11.7 (9.5–13.8)	13.3 (10.9–15.7)	5.2 (4.0–6.2)	25.7 (21.0–30.4)	6.8 (4.9–8.7)
Obesity	14.4 (12.9–15.8)	18.5 (16.6–20.3)	7.9 (6.8–8.8)	34.8 (31.3–38.2)	8.6 (7.2–9.9)
*P* value	0.039	0.002	0.002	0.002	0.122
Hepatic steatosis on ultrasound					
No	13.2 (11.8–14.5)	16.8 (15.1–18.6)	6.8 (5.9–7.7)	30.2 (26.9–33.4)	7.7 (6.6–8.9)
Yes	14.8 (12.1–17.5)	17.7 (14.2–21.1)	7.9 (6.1–9.6)	38.4 (32.2–44.4)	9.0 (6.3–11.8)
*P* value	0.242	0.651	0.254	0.012	0.301

Data are expressed as mean (95% confidence interval). *P* values correspond to independent-samples Student's t-test.

Across all three stratification analyses, *omental* fat was the only compartment to show statistically significant differences between all groups. Males exhibited markedly greater *omental* fat thickness than females (40.5 mm vs. 27.3 mm; *p* < 0.001); patients with obesity had significantly thicker *omental* fat than those with overweight and even overweight with abdominal obesity (34.8 mm vs. 25.7 mm; *p* = 0.002); and patients with hepatic steatosis presented substantially greater *omental* thickness compared to those without steatosis (38.4 mm vs. 30.2 mm; *p* = 0.001).

When comparing obesity versus overweight subgroups, superficial subcutaneous, deep subcutaneous, and *preperitoneal* fat layers were also significantly thicker in patients with obesity (*p* = 0.039, *p* = 0.002, and *p* = 0.002, respectively), whereas right *perirenal* fat did not differ significantly in any of the three comparisons performed. No significant sex-related differences were identified for any compartment other than the *omental* layer, and neither subcutaneous, *preperitoneal* nor *perirenal* fat distinguished patients with from those without hepatic steatosis.

### 3.3 Associations between abdominal fat layers and cardiometabolic risk parameters

**Figure 1:**
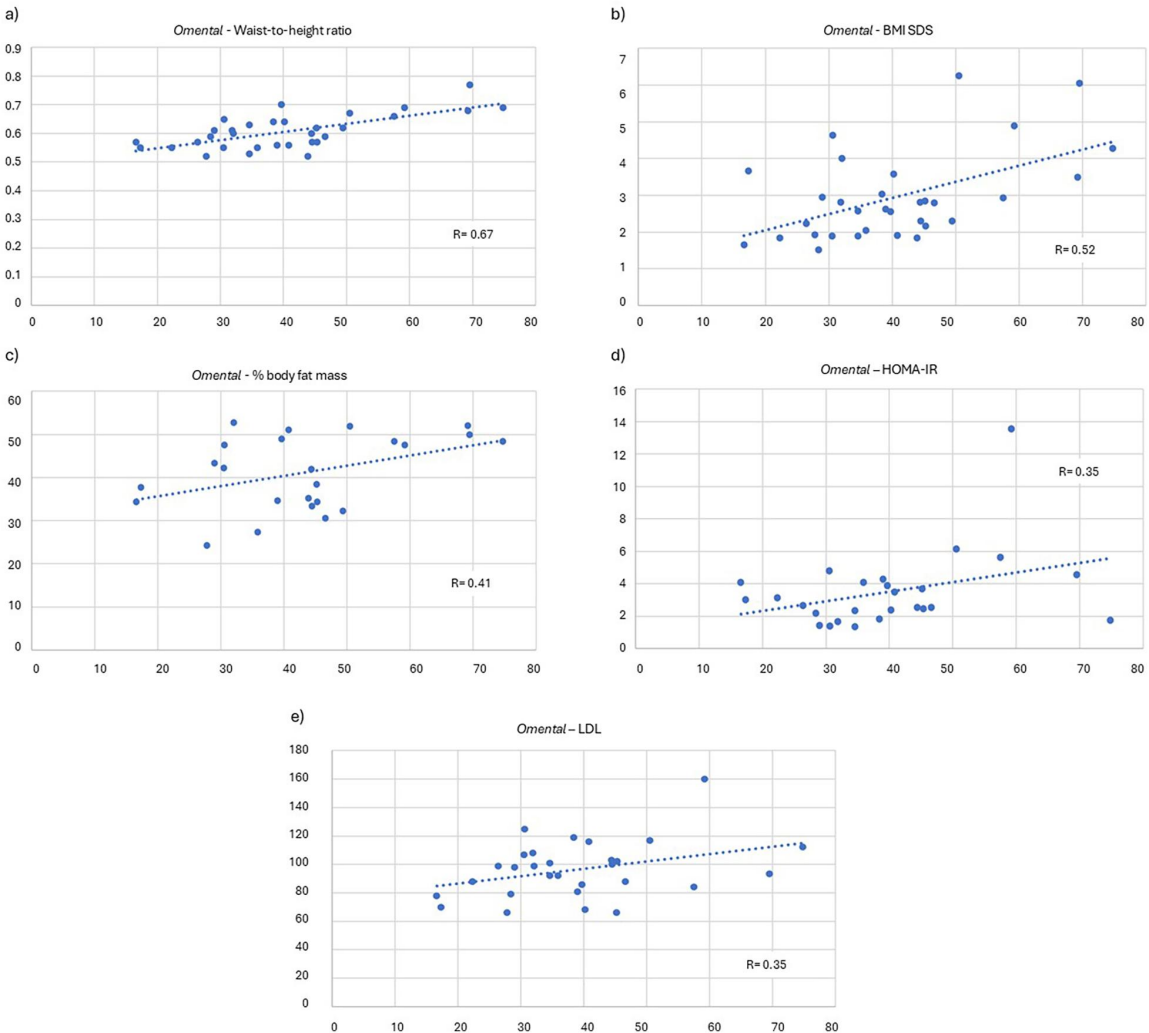
Pearson correlation between *omental* fat thickness and cardiometabolic risk parameters in male pediatric patients with overweight or obesity. BMI, body mass index; HOMA-IR, homeostatic model assessment for insulin resistance; LDL, low-density lipoprotein; SDS, standard deviation score. Scatter plots with the association of *omental* fat thickness (mm, *x*-axis) with **(a)** waist-to-height ratio, **(b)** BMI standard deviation score (SDS), **(c)** body fat percentage, **(d)** HOMA-IR, and **(e)** LDL cholesterol (mg/dL). Pearson correlation coefficients (R) are indicated within each panel. Dashed lines represent the linear regression fit.

In males, *omental* fat demonstrated positive correlations with all three anthropometric indices, with the waist-to-height ratio the strongest association (r = 0.67; Figure 1a), followed by BMI SDS (r = 0.52; Figure 1b) and body fat percentage (r = 0.41; Figure 1c). Positive correlations were also observed in males with HOMA-IR (r = 0.35; Figure 1d) and LDL cholesterol (r = 0.35; Figure 1e), while no clinically meaningful associations were identified with triglycerides (r = 0.05) or HDL cholesterol (r = 0.04) (Table 3).

**Table 3:** Pearson correlation coefficients (r) between *omental* fat layer thickness and cardiometabolic risk parameters, stratified by sex.

Group	Body fat (%)	BMI (SDS)	Waist-to-height ratio	HOMA-ir	LDL (mg/dL)	Triglycerides (mg/dL)	HDL (mg/dL)
*Omental* fat males	0.41	0.52	0.67	0.35	0.35	0.05	0.04
*Omental* fat females	0.29	0.18	0.39	−0.11	0.20	0.07	−0.06

BMI, body mass index; HDL, high-density lipoprotein; HOMA-IR, homeostatic model assessment for insulin resistance; LDL, low-density lipoprotein; SDS, standard deviation score.

**Figure 2:**
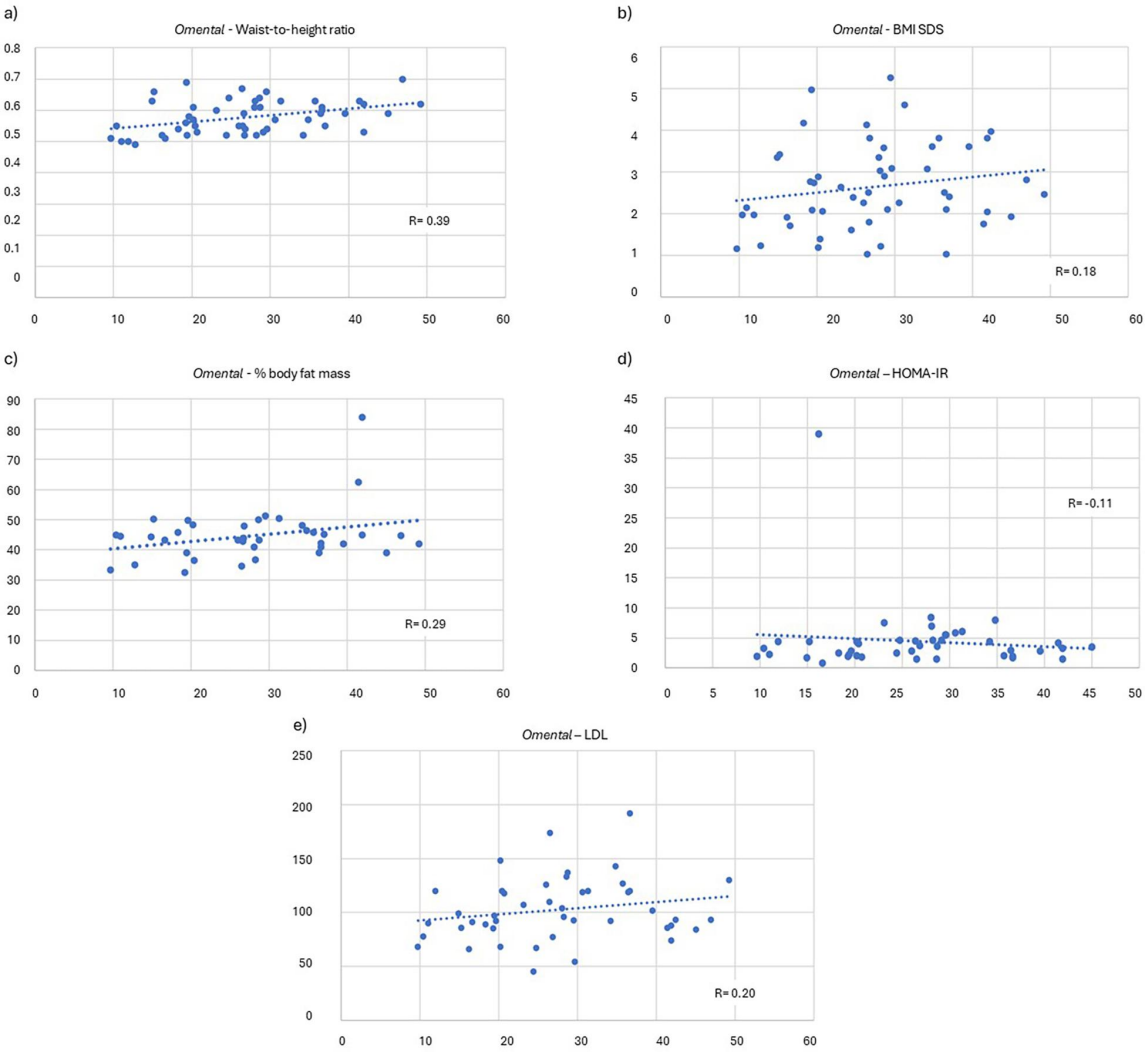
Pearson correlation between *omental* fat thickness and cardiometabolic risk parameters in female pediatric patients with overweight or obesity. BMI, body mass index; HOMA-IR, homeostatic model assessment for insulin resistance; LDL, low-density lipoprotein; SDS, standard deviation score. Scatter plots with the association of *omental* fat thickness (mm, *x*-axis) with **(a)** waist-to-height ratio, **(b)** BMI standard deviation score (SDS), **(c)** body fat percentage, **(d)** HOMA-IR, and **(e)** LDL cholesterol (mg/dL). Pearson correlation coefficients (R) are indicated within each panel. Dashed lines represent the linear regression fit.

In females, the associations with *omental* fat were attenuated across all parameters, with the waist-to-height ratio showing the strongest correlation (r = 0.39; Figure 2a), followed by body fat percentage (r = 0.29; Figure 2c) and BMI SDS (r = 0.18; Figure 2b). No clinically relevant association was observed with HOMA-IR (r = −0.11; Figure 2d), LDL (r = 0.20; Figure 2e), triglycerides (r = 0.07), or HDL cholesterol (r = −0.06) (Table 3).

### 3.4 Comparison of abdominal fat layer thickness between overweight and obesity subgroups

In males, BMI SDS correlated moderately to strongly with all five compartments, with deep subcutaneous fat presenting the strongest association (r = 0.70), followed by superficial subcutaneous (r = 0.61), *perirenal* (r = 0.55), *preperitoneal* (r = 0.53), and *omental* fat (r = 0.52). In females, however, correlations with BMI SDS were smaller across all layers, with deep subcutaneous (r = 0.37) and *preperitoneal* fat (r = 0.36) showing only modest associations, and *omental* fat showing no correlation with BMI SDS (r = −0.18) (Table 4).

**Table 4:** Pearson correlation coefficients (r) between abdominal fat layer thickness and BMI (SDS), stratified by sex.

Group	Superficial subcutaneous fat	Deep subcutaneous fat	*Preperitoneal* fat	*Omental* fat	Right *perirenal* fat
BMI SDS males	0.61	0.70	0.53	0.52	0.55
BMI SDS females	0.21	0.37	0.36	−0.18	0.20

### 3.5 ROC curve analysis and optimal cut-off values for *omental* fat thickness

**Figure 3:**
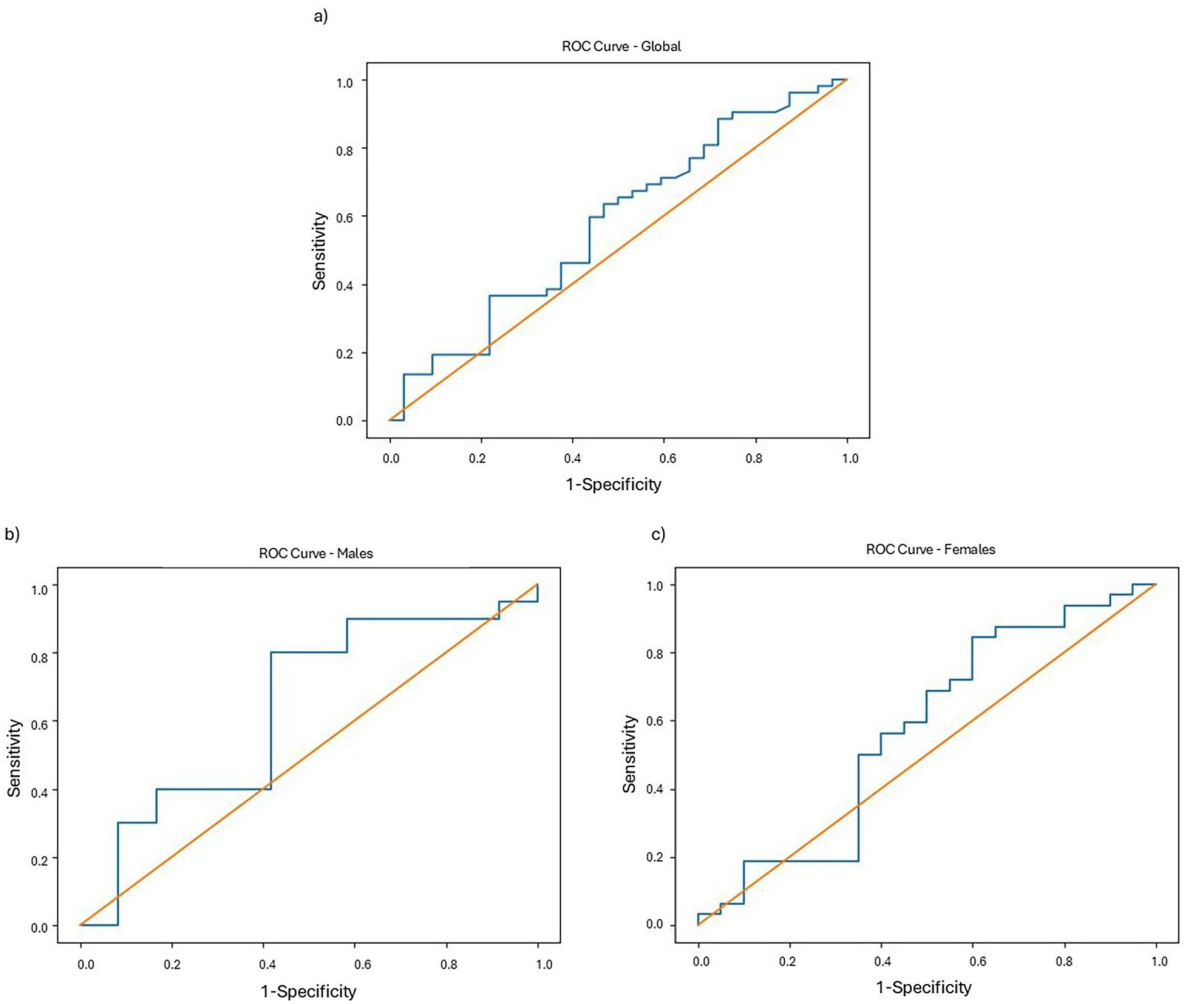
ROC curves for *omental* fat thickness as a discriminator of metabolically unhealthy status in pediatric patients with overweight or obesity. **(a)** Presents the overall cohort; **(b)** males; **(c)** females. The orange diagonal represents the line of no discrimination. Metabolically unhealthy status was defined as the fulfilment of at least one of the following criteria: HDL-cholesterol < 40 mg/dL, triglycerides > 150 mg/dL, fasting plasma glucose > 100 mg/dL, blood pressure above the 90th percentile for age, sex, and height, HOMA-IR > 3.16, or ultrasound-diagnosed hepatic steatosis. Optimal cut-off values were determined by maximization of the Youden index; 95% confidence intervals were estimated by non-parametric bootstrap resampling (2,000 iterations).

In the overall cohort, the AUC was 0.58, with an optimal cut-off of 28.6 mm (sensitivity 63%, specificity 53%; 95% CI of cut-off: 19.3–49.2 mm; Figure 3a). Sex-stratified analyses presented a higher discriminatory performance in males (AUC 0.64), with an optimal cut-off of 34.6 mm (sensitivity 80%, specificity 58%; 95% CI: 30.5–49.4 mm; Figure 3b). In females, the AUC was 0.57, with an optimal cut-off of 19.6 mm (sensitivity 84%, specificity 40%; 95% CI: 15.2–39.5 mm; Figure 3c).

## 4 Discussion

Our results point to the *omental* fat layer as the visceral compartment most consistently associated with cardiometabolic risk markers in childhood and adolescence. Among all the abdominal compartments evaluated, *omental* fat was the only one that consistently associated with metabolic risk markers, distinguished patients with hepatic steatosis from those without it, and showed a clear sexual dimorphism favoring males. More importantly, the data suggests that of all the visceral fat depots known to carry metabolic significance in adult obesity, the *omental* compartment may be the first to exhibit pathological expansion in childhood, although longitudinal studies are required to confirm this temporal sequence. These cross-sectional observations are consistent with an age-dependent pattern of ectopic fat accumulation, with *omental* fat representing the earliest detectable visceral abnormality in the natural history of obesity.

To our knowledge, this is one of the first studies to apply a structured, multi-compartment ultrasound evaluation of abdominal fat layers in a pediatric cohort using the *Eco-Obesity* approach. While this protocol has been validated and mainly applied in adult populations with obesity (5, 7), its extension to children and adolescents has not been previously reported. This represents a significant knowledge gap, as most prior studies in this age group have relied on aggregate measures of visceral adiposity derived from DEXA, computed tomography, or magnetic resonance imaging, none of which can discriminate between the preperitoneal, *omental*, and perirenal compartments individually. The present study therefore provides a novel contribution by demonstrating that ultrasound-based stratification of abdominal fat layers is technically feasible in children as young as 6 years and provides clinically meaningful associations with established metabolic risk markers.

The differential behavior of the five fat compartments across the three stratification analyses performed is consistent with the pattern previously described in adult cohorts using the same *Eco-Obesity* protocol. In our recently published adult data (5), both *omental* and *perirenal* fat correlated strongly with anthropometric indices, DEXA-derived VAT, and metabolic parameters, while preperitoneal fat showed no significant associations. This pattern had already been proposed by Cuatrecasas et al. (7), where sex-specific cut-off values for *omental* fat (54 mm in males/34 mm in females) were established as predictive thresholds for metabolic syndrome-associated cardiovascular risk. The present pediatric cohort recapitulates the metabolic relevance of *omental* fat and the metabolic inertness of *preperitoneal* fat regardless of age, reinforcing the argument that the *preperitoneal* layer does not clearly represent pathological visceral fat and that its associations with cardiovascular risk factors are inconsistent across populations (12, 14). The strong association between *omental* thickness and the presence of hepatic steatosis is in line with previous findings where if was demonstrated that intra-abdominal fat thickness was an effective predictor of Metabolic dysfunction-associated steatotic liver disease (MASLD) (15), and supports the hypothesis that *omental* fat, through its direct portal drainage into the liver, constitutes the primary driver of ectopic hepatic lipid deposition in children with obesity. It should be noted that HOMA-IR, as a surrogate derived from fasting glucose and insulin concentrations, predominantly reflects hepatic rather than whole-body insulin sensitivity. Consequently, the correlation between *omental* fat thickness and HOMA-IR observed in males may be specifically driven by the portal delivery of *omental*-derived free fatty acids to the liver, promoting hepatic lipid accumulation and hepatic insulin resistance, rather than indicating a generalized peripheral insulin-resistant phenotype. This interpretation is consistent with the concurrent association between *omental* fat and ultrasound-diagnosed hepatic steatosis in the present cohort. A notable divergence from the adult reference data of Cuatrecasas et al. (7) is the absence of significant correlations between *omental* fat and the lipid variables triglycerides and HDL cholesterol in the present cohort, in contrast to the significant associations reported for both sexes in the adult series. This discrepancy may reflect a stage-dependent progression in the metabolic consequences of *omental* fat expansion: whilst *omental* adiposity in adults has already exerted a sustained lipotoxic and pro-inflammatory burden on hepatic lipid metabolism sufficient to alter the triglyceride-rich lipoprotein axis and suppress HDL synthesis, the pediatric patients in our cohort may represent an earlier phase in this trajectory, in which portal free fatty acid delivery has not yet reached the threshold required to produce frank dyslipidemia.

The absence of significant differences in right *perirenal* fat thickness between weight status subgroups, and its lack of association with any metabolic parameter evaluated, diverges from what has been consistently observed in adult populations using the same protocol, and warrants specific consideration. A plausible explanation is provided by Bassols et al. (16), who demonstrated in prepubertal Caucasian children that *perirenal* fat was the only abdominal depot independently associated with carotid intima-media thickness, suggesting that this compartment may exert its pathogenic effects in childhood preferentially through vascular rather than metabolic pathways. Since carotid intima-media thickness was not assessed in the present cohort and the primary endpoints were metabolic parameters, the vascular relevance of *perirenal* fat, although not being increased in our patients, cannot be excluded and represents an important topic for future investigation.

The ROC curve analysis provides a preliminary, exploratory attempt at identifying *omental* fat thickness thresholds with clinical applicability in this pediatric age group, although with the caveats inherent to a modestly powered, single-center cohort. The global AUC of 0.58 (optimal cut-off 28.6 mm; sensitivity 63%, specificity 53%) reflects a limited overall discriminatory capacity when discriminating metabolically unhealthy from healthy individuals using the composite definition applied here. The modest overall AUC is consistent with the biological heterogeneity of a mixed-sex sample with a wide age and pubertal range. Sex-stratified analysis, however, revealed a more informative pattern: in males, the AUC of 0.64 and an optimal cut-off of 34.6 mm (sensitivity 80%, specificity 58%; 95% CI: 30.5–49.4 mm) shows a higher and more consistent relationship between *omental* fat and metabolically unhealthy status, concordant with the stronger metabolic correlates of *omental* fat observed in this subgroup. In females, the optimal cut-off of 19.6 mm had a high sensitivity (84%) at the cost of reduced specificity (40%; AUC 0.57; 95% CI: 15.2–39.5 mm), which may reflect the lower absolute burden of *omental* fat in girls relative to boys at equivalent levels of overall adiposity, and suggests that high sensitivity may be the operationally appropriate objective when screening for cardiometabolic risk in female adolescents. The sex-specific cut-offs identified in this pediatric cohort (34.6 mm in males; 19.6 mm in females) are considerably lower than those previously established in adults using the same *Eco-Obesity* protocol (54 mm in males; 34 mm in females) (7), suggesting that pathological *omental* increase in children occurs at earlier, lower absolute thresholds, and that adult-derived reference values are not directly transferable to pediatric populations. These sex-specific cut-off values should be considered hypothesis-generating and require validation in independent, larger cohorts before any clinical application can be considered.

The marked sexual dimorphism in *omental* fat thickness and its metabolic correlates observed in the present cohort is consistent with established evidence on the role of androgens in promoting visceral fat accumulation during puberty. The finding that *omental* fat correlated with HOMA-IR, LDL cholesterol, and all three anthropometric indices in males, whereas these associations were systematically absent or attenuated in females, aligns with previous observations which reported no significant sex differences in intra-abdominal fat associations in a prepubertal children (17), in contrast to the dimorphism documented here in a predominantly pubertal sample. This temporal contrast supports the hypothesis that androgenic stimulation during male puberty is the primary driver of the divergence between sexes in *omental* fat deposition and its metabolic consequences, instead of a constitutive biological difference present from early life. Furthermore, the absence of a meaningful correlation between BMI SDS and *omental* fat in females, in contrast to the moderate correlation observed in males, has direct clinical implications. In female adolescents with obesity, BMI SDS alone is insufficient to identify those with pathological *omental* fat expansion, reinforcing the added diagnostic value of structured ultrasound evaluation of visceral fat compartments over conventional anthropometric surrogates in this subgroup. These sex-specific patterns are consistent with the metabolomic findings reported in the literature (18), who demonstrated significant sex differences in biomarkers associated with insulin resistance in obese adolescents at comparable BMI z-scores, with males exhibiting a distinct metabolic profile characterized by higher branched-chain amino acid and acylcarnitine levels, supporting the notion that sex modulates the metabolic consequences of excess adiposity independently of the degree of overall obesity.

The present study has several methodological strengths. The use of a standardized, multi-compartment ultrasound protocol applied by a single experienced operator minimizes intra-observer variability and ensures the comparability of measurements across patients; furthermore, the inclusion of five distinct fat compartments provides a level of anatomical resolution not achievable with conventional DEXA-based or anthropometric approaches, and the simultaneous assessment of multiple cardiometabolic risk domains, including anthropometric indices, insulin resistance, lipid profile, and hepatic steatosis, allows for a comprehensive characterization of the metabolic correlates of each depot. The principal limitation of this work is its retrospective, single-centre design with a relatively modest sample size (*N* = 84), which constrains statistical power, particularly for sex-stratified subgroup analyses, and precludes the establishment of causal or longitudinal relationships between fat depot expansion and metabolic deterioration. The absence of carotid intima-media thickness measurements represents a specific gap that limits the assessment of the vascular relevance of *perirenal* fat in this cohort, although this fat layer has not been shown to increase in our population. Finally, unlike the adult *Eco-Obesity* protocol, EAT was not assessed echocardiographically in this cohort, which limits a full characterization of the visceral fat phenotype given the established associations between epicardial fat and cardiometabolic risk markers. Additionally, body fat percentage was estimated by bioelectrical impedance rather than by DEXA, which introduces a potential source of measurement error, particularly in patients with high degrees of adiposity where impedance-based models may be less accurate. Finally, the predominantly pubertal and female composition of the cohort, reflecting the referral patterns of the participating unit, may limit the generalizability of the findings to prepubertal children and to population-based samples with a more balanced sex distribution. The cross-sectional nature of this investigation precludes the establishment of temporal or causal relationships between *omental* fat expansion and metabolic deterioration; the associative patterns described herein should therefore be interpreted as hypothesis-generating and require confirmation in prospective longitudinal studies. The analytical approach was restricted to univariate comparisons and bivariate correlations without adjustment for potential confounders such as age, BMI z-score, and pubertal status; although this was consistent with the exploratory objectives and sample size constraints, it represents a methodological limitation. The composite definition of metabolically unhealthy status, based on the presence of at least one abnormal metabolic parameter, creates a heterogeneous outcome that may reduce the specificity of the classification; future studies with larger sample sizes should consider sensitivity analyses using stricter criteria. The absence of formal adjustment for multiple comparisons across the several fat compartments and metabolic outcomes evaluated increases the risk of type I error, and statistically significant associations should be interpreted with caution. The recruitment of participants from a specialized tertiary care pediatric obesity unit may introduce referral bias, limiting the direct generalizability of the findings to broader pediatric populations. Although pubertal status was recorded for all participants (85.7% pubertal, 61.9% at Tanner stage V), the sample size precluded meaningful stratification by individual Tanner stage. This represents an additional limitation, as pubertal maturation is a well-established modulator of body fat distribution and insulin sensitivity, and the observed associations between *omental* fat and cardiometabolic parameters may be partially confounded by maturational differences. Future studies with larger cohorts should incorporate pubertal stage-stratified or pubertal stage-adjusted analyses to disentangle the independent contribution of visceral fat expansion from that of pubertal development. Data on gestational age and birth weight were not available for the present cohort. Given the established role of perinatal factors, including low birth weight and prematurity, as determinants of abdominal fat distribution and metabolic risk in later life, and the potential for sex-specific differences in these early-life exposures, the absence of this information represents a limitation that should be addressed in future prospective studies.

The present findings should not be interpreted as evidence of a causal relationship between *omental* fat expansion and metabolic deterioration. Several well-established observations challenge causal models linking visceral adiposity to metabolic disease. Youth-onset type 2 diabetes is approximately twice as common in adolescent females as in males in Western populations, despite females accumulating less visceral fat; African American individuals exhibit lower visceral adipose tissue mass and lower rates of metabolic dysfunction-associated steatotic liver disease than Caucasian individuals, yet carry a disproportionately higher burden of type 2 diabetes; and a randomized controlled trial (19) demonstrated that surgical removal of *omental* fat did not improve insulin sensitivity or cardiovascular risk factors in obese adults. These observations indicate that the pathogenesis of insulin resistance and type 2 diabetes is multifactorial and population-specific, involving genetic susceptibility, hormonal factors, pancreatic beta-cell reserve, and tissue-specific insulin sensitivity determinants that act independently of visceral fat volume. Also, the metabolic behavior of actively expanding *omental* adipose tissue in pediatric obesity, characterized by adipocyte hypertrophy and pro-inflammatory secretion, may differ fundamentally from the chronically expanded and fibrotic *omental* tissue present in adults undergoing bariatric surgery, where metabolic damage may have become self-sustaining through pathways no longer dependent on continued portal free fatty acid delivery. The associations reported here should therefore be understood as correlational and stage-specific, and not as indicators of a direct or unidirectional causal mechanism.

## 5 Conclusions

The present study demonstrates that structured ultrasound assessment of abdominal fat layers using the *Eco-Obesity* protocol is technically feasible and clinically informative in pediatric patients with obesity and overweight, providing a level of visceral fat compartmentalization not achievable with conventional anthropometric or DEXA-based approaches. Among all five compartments evaluated, the *omental* fat layer was as the only depot consistently associated with an anthropometric obesity index, insulin resistance, LDL cholesterol, and hepatic steatosis, and was the only one to demonstrate a statistically significant sexual dimorphism favoring males, while subcutaneous, *preperitoneal*, and *perirenal* compartments remained largely quiescent from a metabolic standpoint at this stage of life. In children, routine ultrasound assessment may focus solely on *omental* fat, streamlining the overall evaluation. These findings support the hypothesis that *omental* fat may represent the visceral depot most closely linked to early metabolic dysfunction in pediatric obesity, although the cross-sectional design precludes definitive conclusions regarding temporal sequence or the order of metabolic involvement of *perirenal* and other compartments that characterizes the adult disease phenotype. The waist-to-height ratio was identified as the anthropometric parameter most closely correlated with *omental* fat thickness in both sexes, reinforcing its clinical utility as a screening tool, while the systematic attenuation of *omental* fat associations in females highlights the necessity of sex-stratified interpretation of visceral adiposity assessments in pubertal populations.

Future investigations should prioritize the validation in a larger population of the sex- and age-specific *omental* fat thickness cut-off values represent an immediate and clinically actionable research priority in pediatric endocrinology.

## Data Availability

The raw data supporting the conclusions of this article will be made available by the authors, without undue reservation.
